# Three nervous system-specific expressed genes are potential biomarkers for the diagnosis of sporadic amyotrophic lateral sclerosis through a bioinformatic analysis

**DOI:** 10.1186/s12920-023-01441-x

**Published:** 2023-01-27

**Authors:** Yifu Liao, Haiping Cai, Feifei Luo, Dongcheng Li, Hao Li, Geng Liao, Jinhai Duan, Renshi Xu, Xiong Zhang

**Affiliations:** 1 Department of Neurology, Guangdong Provincial People’s Hospital (Guangdong Academy of Medical Sciences), Southern Medical University, Guangzhou, China; 2 Department of Neurosurgery, Guangdong Provincial People’s Hospital (Guangdong Academy of Medical Sciences), Southern Medical University, Guangzhou, China; 3 Cancer Epigenetics Laboratory, Department of Clinical Oncology, State Key Laboratory of Oncology in South China, Sir YK Pao Center for Cancer and Li Ka Shing Institute of Health Sciences, The Chinese University of Hong Kong, Shatin, Hong Kong; 4 The First School of Clinical Medicine, Southern Medical University, Guangzhou, China; 5 Eastern Department of Neurology, Guangdong Geriatrics Institute, Guangdong Provincial People’s Hospital (Guangdong Academy of Medical Sciences), Southern Medical University, Guangzhou, China; 6 Department of Neurology, Jiangxi Provincial People’s Hospital, The First Affiliated Hospital of Nanchang Medical College, Clinical College of Nanchang Medical College, Nanchang, China; 7 Department of Neurology, Maoming People’s Hospital, Maoming, China

**Keywords:** Sporadic amyotrophic lateral sclerosis, Biomarker, Competitive endogenous RNA, Single nucleotide polymorphism, Bioinformatic analysis

## Abstract

**Background:**

Amyotrophic lateral sclerosis (ALS) is the most common neurodegenerative disease in adults. However, ALS, especially sporadic ALS (sALS), is difficult to diagnose due to the lack of biomarkers.

**Results:**

We used the bioinformatics technology to find the potential biomarker and we found that two hundred seventy-four DEGs were identified and enrichment analysis showed DEGs were involved in nervous system activity, like axon_guidance and the neurotrophin_signaling_pathway. Five nervous system-specific expressed hub genes were further validated by three GEO datasets. APP, LRRK2, and PSEN1 might be potential diagnostic and prognostic biomarkers of sALS, and NEAT1-miR-373-3p/miR-302c-3p/miR-372-3p-APP, circ_0000002-miR-302d-3p/miR-373-3p-APP and XIST-miR-9-5p/miR-30e-5p/miR-671-5p might be potential ceRNA regulatory pathways. APP SNP analysis showed subjects harboring the minor G allele of rs463946, minor G allele of rs466433 and minor C allele of rs364048 had an increased risk of sALS development.

**Conclusions:**

Our results identified three nervous system-specific expressed hub genes that might be diagnostic and prognostic markers of sALS and APP might be a genetic susceptibility factor contributing to sALS development.

**Supplementary Information:**

The online version contains supplementary material available at 10.1186/s12920-023-01441-x.

## Background

Amyotrophic lateral sclerosis (ALS) is a progressive and aggravated neurodegenerative disease and the most common motoneuron disease in adults. ALS is characterized by the progressive degeneration of upper and lower motor neurons in the cerebral cortex, brain stem and anterior horn of the spinal cord, which leads to amyotrophy of the limbs and trunk, and eventually, patients die because of respiratory failure caused by predominant diaphragm dysfunction [1]. The clinical characteristics of ALS patients are the coexistence of symptoms and signs of upper and lower motor neuron damage, featuring as different combinations of muscle weakness, amyotrophy and pyramid signs [2]. ALS has a prevalence of 1–2 per 100,000 people worldwide, and the average age of onset is approximately 55 years [3]. The clinical heterogeneity among ALS patients leads to difficulty in diagnosis, and currently, definitive diagnostic tests are lacking [4]. In addition, effective treatments for ALS are not available since riluzole and edaravone, two FDA-approved drugs for ALS treatment, only delay the progression of ALS in some patients, and therapeutic interventions are still based on symptom management and respiratory support [5, 6]. The average survival time of ALS patients is 3–5 years [7]. Therefore, a detailed understanding of ALS development could help with more effective intervention in the early stages of the disease. Based on the family history, patients are divided into familial ALS (fALS) and sporadic ALS (sALS) [8]. Genetic disorders are considered an important cause of ALS, and several known genes and/or loci have been reported to affect the development of fALS, such as SOD1, FUS and C9orf72 [9–11]. Because several genes/loci involved in sALS development have been identified, sALS is considered to have a genetic basis and more complex pathogenesis [12]. The biomarkers investigation of sALS has been ongoing for many years, however, effective biomarkers and exact genetic mechanism of sALS still need further studied [13].

Transcriptomics and microarray analyses are important techniques in disease research and have been widely used to identify novel biomarkers and improve the diagnosis and treatment of various diseases, such as tumors and neurodegenerative diseases [14, 15]. Competing endogenous RNAs (ceRNAs) can competitively bind miRNAs through microRNA response elements (MREs) to regulate the expression level of each other, forming a large-scale gene expression regulatory network in the transcriptome [16, 17]. The ceRNA regulatory network plays an important role in the occurrence and development of neurodegenerative diseases [18]. Therefore, it is possible to explore potentially key genes and pathway networks closely related to disease development through a combination of microarray and bioinformatical technologies.

In the present study, we performed a bioinformatics analysis using publicly available gene expression datasets to search for sALS susceptibility genes. In addition, we conducted a case–control study including 30 sALS patients and 30 nonneurological controls in the Chinese Han population. We detected single nucleotide polymorphisms (SNPs) of the susceptibility gene risk fragments to verify the relationship between these SNPs of susceptibility genes and sALS. We aimed to identify some diagnostic biomarkers in the early stage of sALS development.

## Methods

### Gene expression data acquisition

The microarray data, which were samples (nervous tissues, muscular tissues or whole blood) of sALS patients, were obtained from the Gene Expression Omnibus (GEO) database. Six GEO datasets and eight GPL platforms were included in our study, including GSE833, GSE26276, GSE4595, GSE26927, GSE39644, GSE112681, GPL80 (Affymetrix), GPL6244 (Affymetrix), GPL1708 (Agilent-012391), GPL6255 (Illumina humanRef-8), GPL10558 (Illumina HumanHT-12), GPL15846/GPL15847 (NanoString Technologies) and GPL6947 (Illumina HumanHT-12). We divided these datasets into the test set and the validation set. The test set included GSE833 and GSE26276, including 11 samples (sALS, nonneurological control, and fALS) and 9 samples (sALS, multifocal motor neuropathy [MND], and nonneurological control), respectively. The mRNA expression data were acquired from tissue specimens of the spinal cord and skeletal muscle. The validation set included GSE4595, GSE26927, GSE39644 and GSE112681, which included 20 samples (sALS and nonneurological control), 118 samples (Alzheimer’s disease, sALS, fALS, Huntington’s disease, multiple sclerosis, Parkinson’s disease, nonneurological control and schizophrenia), 48 samples (sALS, fALS, nonneurological control, and multiple sclerosis), and 1117 samples (sALS, fALS and nonneurological control), respectively. The mRNA expression data were acquired from tissue specimens of the motor cortex, spinal cord and whole blood. An expression matrix of nonneurological controls and sALS patients was acquired online. In total, the data of 681 nonneurological controls and 436 ALS patients were analyzed in our study (Table 1). The accessed date of the database was March. 3, 2022.

**Table 1:** Information of selected GEO datasets

GEO accession	Platform	Sample		Age		Sex (male/femal)		Attribute
		Health	sALS	Health	sALS	Health	sALS	
GSE833	GPL80	4	5	–	–	–	–	Test set
GSE26276	GPL6244	3	3	–	–	–	–	Test set
GSE4595	GPL1708	9	11	–	–	–	–	Validation set
GSE26927	GPL6255	10	10	66.8 ± 16.7	68.2 ± 7.6	10/0	7/3	Validation set
GSE39644	GPL15846	10	10	56 ± 11.5	60.1 ± 8.1	3/7	7/3	Validation set
GSE112681	GPL6947&GPL10558	645	397	–	62.0 ± 12.2	357/288	239/158	Validation set

*sALS* Sporadic amyotrophic lateral sclerosis

### Data processing and identification of differentially expressed genes (DEGs)

The raw data downloaded from the GEO database were normalized by the Robust Multiarray Average (RMA) method using the R software (Version 4.1.0) affy package and further transformed into fragments per kilobase of sequence per million mapped reads (FPKM) values for the analysis. The gene expression analysis and analysis of intersample differences were conducted using the limma package. The significance of False Discovery Rate (FDR) q < 0.05. The screening criteria were as follows: Log2 (fold change) > 1.5 or < − 1.5 and an adjusted *p* value ≤ 0.05.

### DEG visualization analysis

Heatmaps and volcano plots were used for the DEG visualization analysis. Briefly, a heatmap was generated with the pheatmap package, and a volcano plot was generated with the ggpubr package by R software.

### Tissue/organ-specific gene expression determination

We determined the tissue/organ-specific expressed genes of the DEGs using the online tool BioGPS (http://biogps.org/). Briefly, the tissue distribution of the DEGs was analyzed, and the screening criteria for tissues/organ-specific genes were as follows: (1) the gene expression location matched a single organ system, and its expression value was greater than 10 times the median value, and (2) the second most abundantly expressed tissues were no more than one-third of the most expressed tissues.

### Performance of the enrichment analysis

A gene set enrichment analysis (GSEA) is a computational method that can be used to determine the distribution trend of genes of interest or concordant differences between two biological states. For the GSEA, we downloaded the GSEA software (version 3.0) and c2: GO gene sets (c2.cp.kegg.v7.4symbols.gmt) to evaluate the pathways and molecular mechanisms of the DEGs. For the GO (Gene Ontology) enrichment analysis, we used the GO annotation of genes in the R software org.Hs.eg.db (version 3.1.0) package and mapped the DEGs onto the background set. The gene enrichment results were analyzed by R software by setting the minimum gene set as 5 and the maximum gene set as 5000. A Q value < 0.05 and an FDR < 0.25 were considered statistically significant. The online database KOBAS 3.0 (http://kobas.cbi.pku.edu.cn/kobas3) was used for the KEGG (Kyoto Encyclopedia of Genes and Genomes) analysis of the DEGs. The use of KEGG data were approved by the Kanehisa laboratory [19].

### PPI network construction

All DEGs were analyzed using the online tool STRING (https://string-db.org/), and a PPI network was constructed under a filter condition of a combined score > 0.4. The interaction information of all DEGs was downloaded and further modified by Cytoscaple software (v3.9.1) for better visualization. The significant gene clusters and related cluster scores were identified by Minimal Common Oncology Data Elements (MCODE) with the following filter criteria: code score cutoff = 0.2; degree cutoff = 2; k core = 2; and maxdepth = 100. CytoHubba is commonly used for significant gene identification (hub genes), and the top 14 hub genes in the DEG network were determined by five algorithms, namely, maximal clique centrality (MCC); degree; density of maximum neighborhood component (DMNC); maximum neighborhood component (MNC); and clustering coefficient. The final hub genes were determined by intersecting all results.

### Prediction of the target miRNAs of the DEGs

The target miRNAs of the hub genes were predicted by the following five online miRNA databases: miRWalk, miRDB, TargetScan, DIANA-micro and miRcode. MiRNAs that were found in at least four databases were selected as the target miRNAs, and a visual messenger RNA (mRNA)–miRNA coexpression network was constructed according to the targeting relationship between the mRNAs and miRNAs using Cytoscape software.

### CeRNA network construction

LncRNAs and circRNAs that interacted with the selected miRNAs were predicted by the online database StarBase (version 3.0) (http://starbase.sysu.edu.cn/index.php). The mRNA–miRNA–lncRNA/circRNA (ncRNAs) interaction was further used for the ceRNA network construction by Cytoscape software.

### Genomic DNA extraction and polymerase chain reaction (PCR)

Samples were collected from sALS patients and nonneurological controls, and the relevant genomic DNA was extracted using a QIAamp DNA Micro Kit (56304, Qiagen, Germany) according to the manufacturer’s instructions. Genomic DNA was further used to perform PCR according to the manufacturer’s instructions. The amplification conditions were as follows: 95 °C predenaturation for 3 min, 94 °C denaturation for 20 s, 58 °C annealing for 20 s, and 72 °C extension for 40 s for 35 cycles. The sequences of the primers are shown in Additional file 1: Table S1.

### Statistical analysis

The statistical analysis was performed using the R software (Version 4.1.0). The continuous variables were compared between the groups using Student’s t-test and presented as the mean ± standard deviation (S.D.) or standard error (S.E.M.). The Kaplan–Meier method was used for the survival analysis, and the difference between the groups was analyzed by the log-rank test. ROC curves were generated using the R software pROC package (version 1.17.0.1), and the AUCs were determined accordingly. A *p* value < 0.05 was considered statistically significant. *p < 0.05; **p < 0.01; ***p < 0.001.

## Results

### Identification of DEGs

**Fig. 1:**
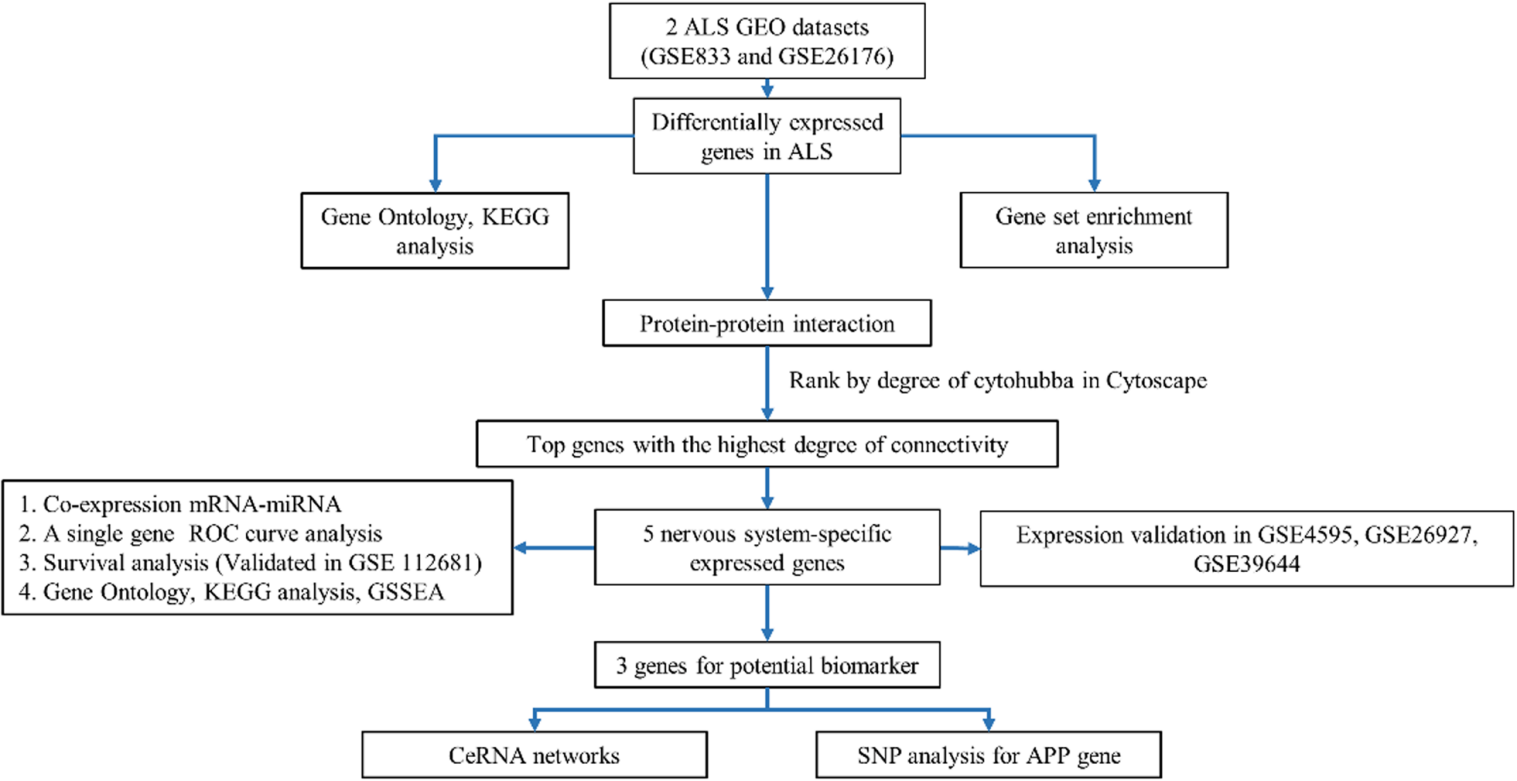
The flow diagram of the study

**Fig. 2:**
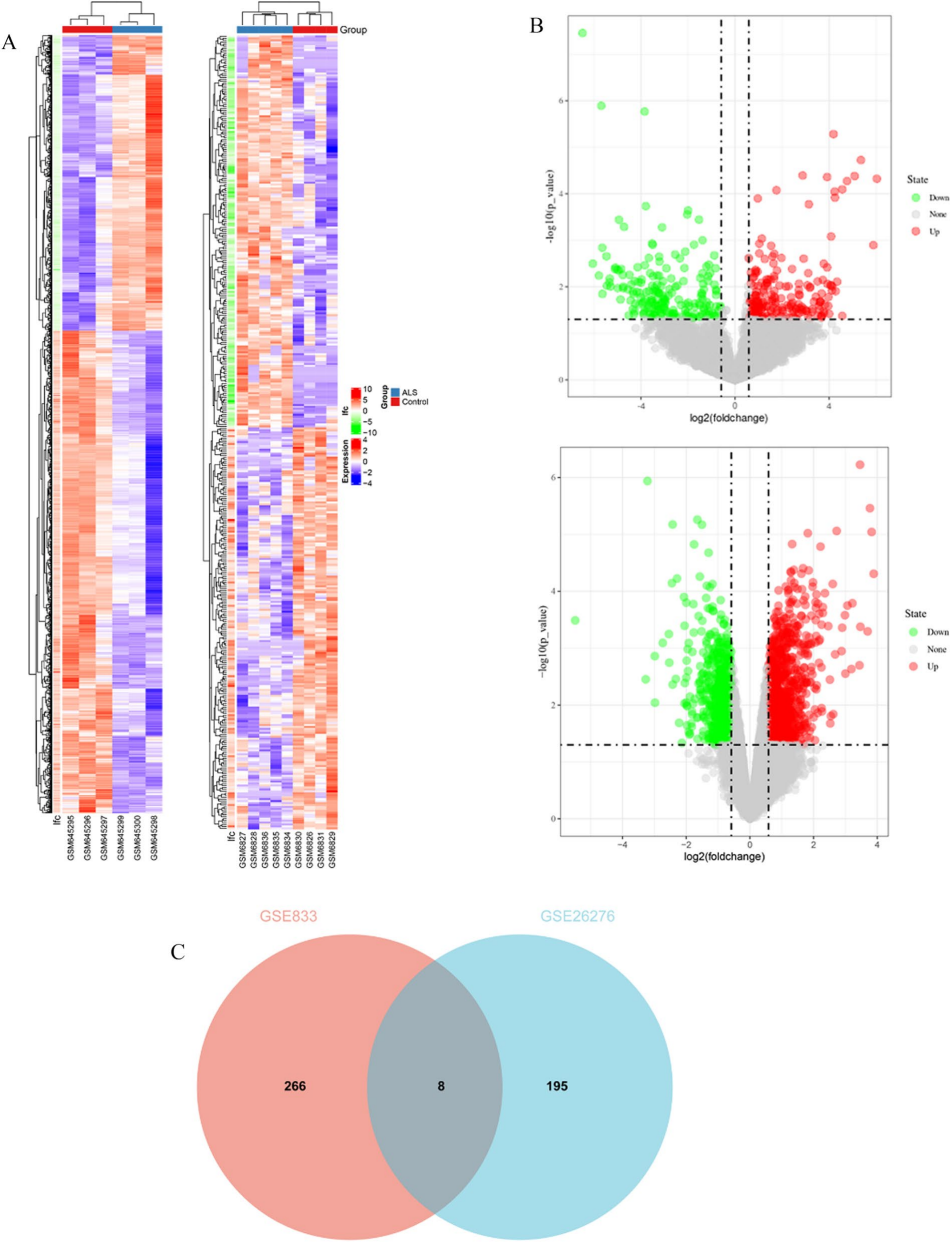
Identification of DEGs. **A** Heatmap of DEGs between sALS samples and nonneurological control samples based on GSE833 and GSE26276. Red rectangles and blue rectangles represent high and low expression, respectively. **B** Volcano plot of DEGs between sALS samples and nonneurological control samples based on GSE833 and GSE26276. Red plot, green plot and gray plot represent upregulated genes, low upregulated genes and nonsignifcant genes, respectively.** C** Eight DEGs were found both in GSE833 and GSE26276

The workflow of this study was shown in Fig. 1. Then, two GEO datasets, GSE833 and GSE26276, in our test set were selected to identify the DEGs. GSE833 included 4 nonneurological control samples and 5 sALS samples, while GSE26276 included 3 nonneurological control samples and 3 sALS samples. A heatmap and volcano plot analysis of two datasets were used to visualize the DEGs (Fig. 2A, B). In the GSE833 dataset, 274 DEGs were identified in the sALS group compared with the nonneurological control samples, including 117 upregulated genes and 157 downregulated genes. In the GSE26276 dataset, 203 DEGs were identified in the sALS group compared with the nonneurological control samples, including 142 upregulated genes and 61 downregulated genes. The Venn plot showed that 8 DEGs were found in both datasets, and we merged the DEGs in the two datasets into a new expression matrix for further analysis (Fig. 2C).

### Analysis of DEG expression in tissues/organs

According to the localization in the gene expression analysis, 46 tissue/organ-specific expressed genes were identified by BioGPS (Table 2). The results showed that most of these DEGs were specifically expressed in the nervous system (24/46, 52.17%). The second tissue/organ-specific expressed system was hematologic/immune cells, including 9 DEGs (9/46, 19.57%), followed by the digestive system (4/46, 9.70%), circulatory system (2/46, 4.35%), endocrine system (2/46, 4.35%), genital system (2/46, 4.35%), respiratory system (1/46, 2.17%), placental system (1/46, 2.17%) and other systems (1/46, 2.17%).

**Table 2:** Tissues/organ-specific DEGs expression

System/organ	Genes	Counts
Haematologic/immune cells	UBE3A, SELL, PTGDR, PRKCD, ARHGAP25, FZR1, DARS1, LARP4B, NUCB2	9
Nervous	APP, PSEN1, SLCO1A2, LRRK2, AKT1, CHRNA4, GDF10, ALK, FHL3, ABCE1, PLP1, LMO3, TF, PRG4, CHI3L1, PDE6H, HTR2A, VAMP1, RXRG, PDE6A, EDNRB, HIP1, OPCML, PUM2	24
Digestive	FABP6, MEP1A, RARRES2, C4BPA	4
Respiratory	F3	1
Circulatory	PGAM2, TNNC	2
Placenta	LGMN	1
Endocrine	CDH1, SERPINA3	2
Genital	SYCP2, SPAG11A	2
Others	GNAT1	1

*DEGs* differentially expressed genes

### DEG enrichment analysis

**Fig. 3:**
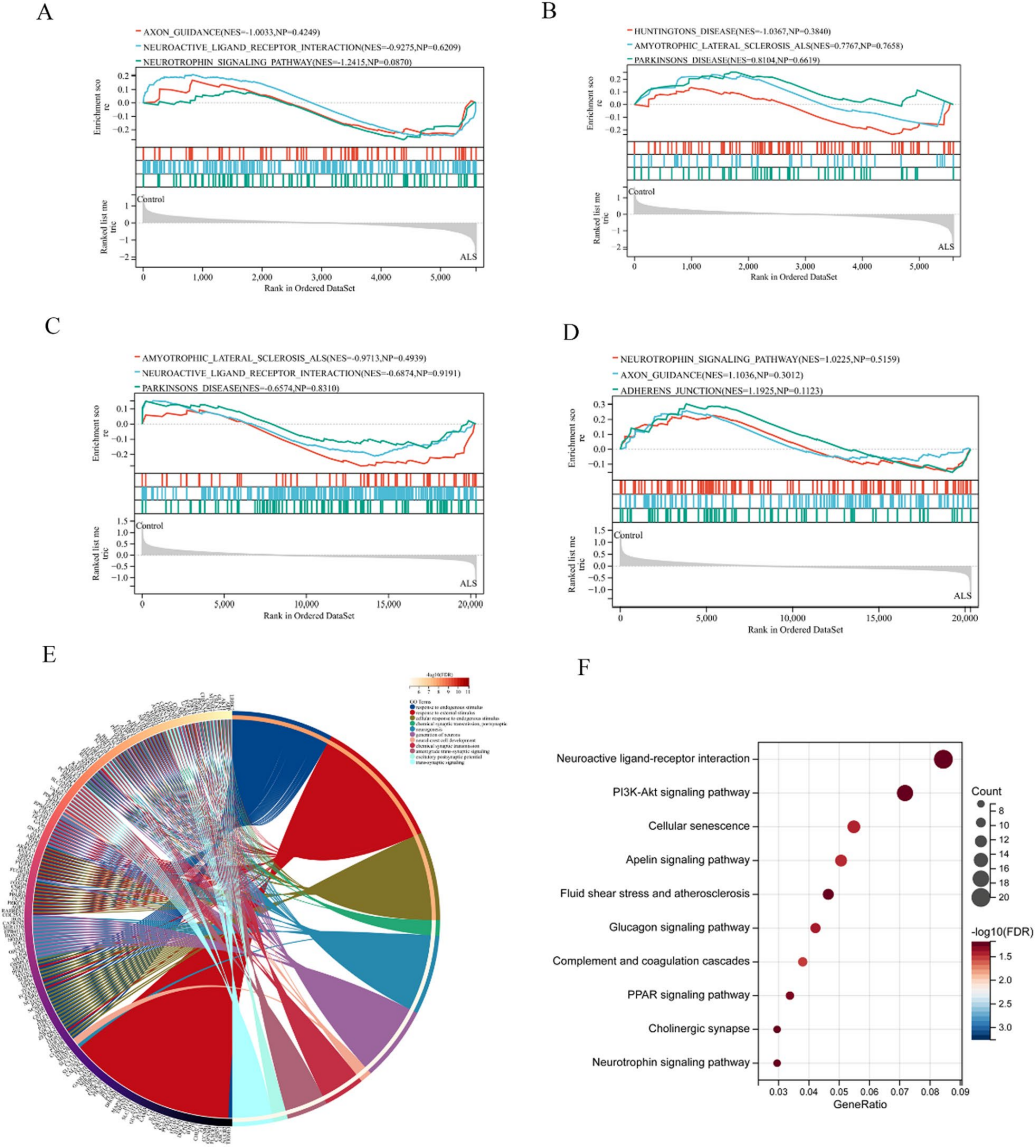
DEG enrichment analysis. **A** and **B** GSEA analysis based on the expression profiles of GSE833. **C** and **D** GSEA analysis based on the expression profiles of GSE26276. **E** The chord plot showed the top 11 enriched biological processes of DEGs. **F** The bubble plot showed the most enriched KEGG signaling pathway

Functional and pathway enrichment analyses of the DEGs were performed by GSEA software, the R software org.Hs.eg.db package and the Online database KOBAS 3.0. First, we performed a GSEA by uploading the expression profiles of GSE833 and GSE26276, and we used the c2: GO gene set to investigate the GO enrichment of gene expression at the overall level. The screening criteria for the significantly enriched gene set were a Q value < 0.05 and an FDR < 0.25. We found that the most enriched gene sets in GSE833 and GSE26276 were related to axon_guidance, neurotrophin_signaling_pathway, neuroactive_ligand_receptor_interaction, adherens_junction, huntingtons_disease, amyotrophic_lateral_sclerosis_als and parkinsons_disease (Fig. 3A–D).

Second, we performed GO and KEGG pathway analyses of the DEGs using the R software org.Hs.eg.db package and KOBAS 3.0, respectively. The results of the GO enrichment analysis of the DEGs showed that processes of nervous system activity, such as chemical synaptic transmission, neural crest cell development and neurogenesis, were significantly related to sALS, and other biological processes, such as response to endogenous stimulus, anterograde transsynaptic signaling and excitatory postsynaptic potential, were also involved. The top 10 biological processes were selected based on a Q value < 0.05 and an FDR < 0.25 and are shown in Fig. 3E. The KEGG pathway analysis revealed that the DEGs were mainly enriched in neuroactive ligand receptor interaction. In addition, the PI3K-Akt signaling pathway, cellular senescence, the apelin signaling pathway, fluid shear stress and atherosclerosis were enriched in the sALS samples (Fig. 3F).

### PPI network analysis and hub gene identification

**Fig. 4:**
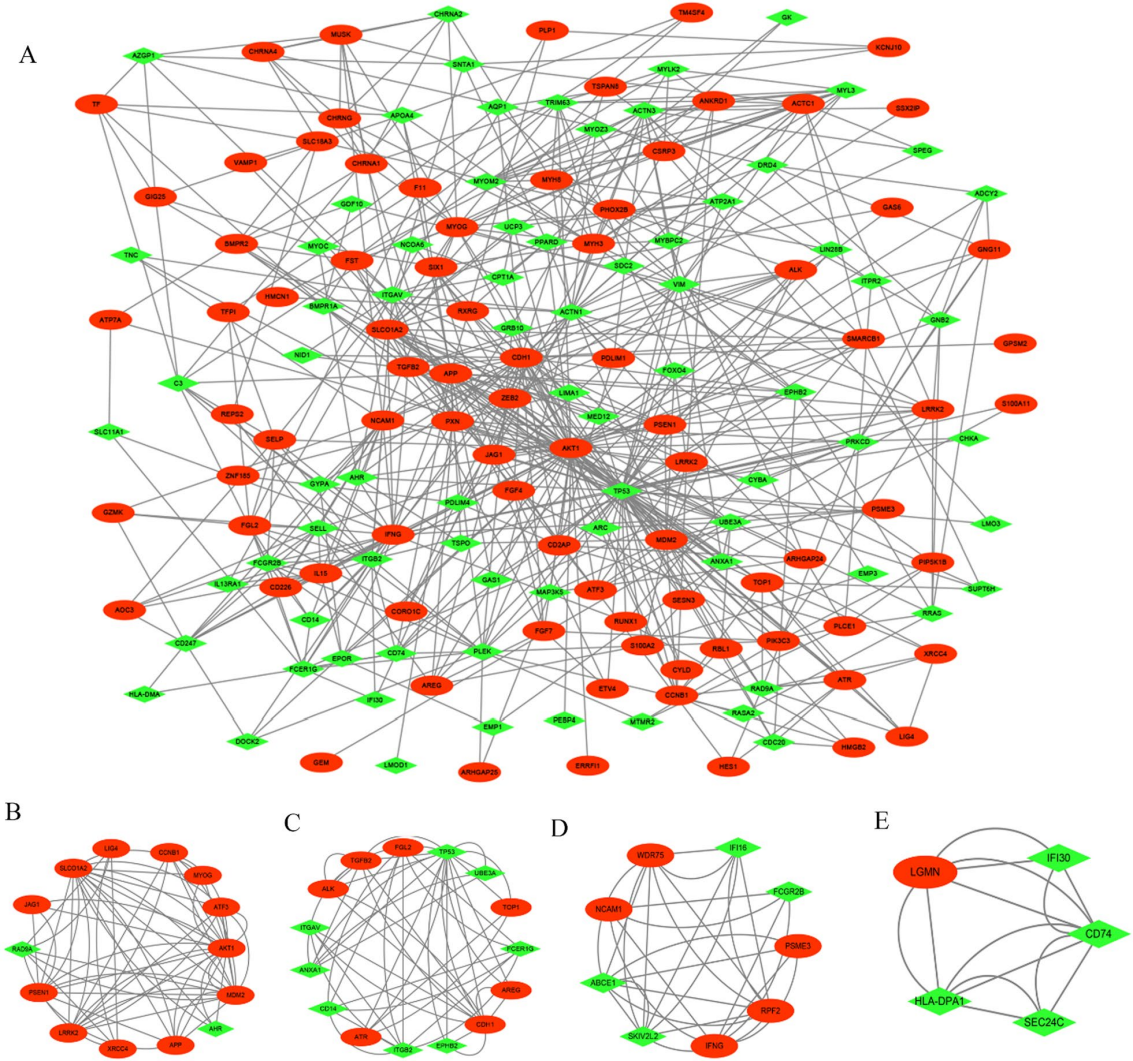
PPI network analysis and hub gene identification. **A** The interaction network of DEGs was comprised 161 nodes and 617 edges. Nodes represent protein and edge represent protein and protein interaction. Red circles represent the upregulated genes and green diamonds represent downregulated genes. **B**–**E** Four cluster modules identified by MCODE. Cluster 1 had 14 nodes and 60 edges with a score of 4.615. Cluster 2 had 15 nodes and 50 edges with a score of 3.571. Cluster 3 had 9 nodes and 28 edges with a score of 3.5 and Cluster 5 nodes and 14 edges with a score of 3.5

The interaction network of proteins coded by the DEGs between the nonneurological controls and sALS patients comprising 161 nodes and 617 edges was evaluated by STRING and visualized by Cytoscape (Fig. 4A). We further used the MCODE plugin to identify the gene cluster modules according to the filter criteria, and the results showed that four modules were identified (Fig. 4B–E). Cluster 1 had 14 nodes and 60 edges with a score of 4.615. Cluster 2 had the second highest cluster score (score: 3.571, 15 nodes and 50 edges), followed by Cluster 3 (score: 3.5, 9 nodes and 28 edges) and Cluster 4 (score: 3.5, 5 nodes and 14 edges). To identify the hub genes in the interaction network, the CytoHubba plugin was used, and the results showed that 14 hub genes were identified by five algorithms of cytoHubba, including Clustering Coefficient, Degree, MNC, MCC and DMNC (Table 3). These DEGs are the core genes in the PPI network, implying that they play an important role in the pathogenesis of sALS. Since the GO, KEGG and GSEA enrichment analyses revealed an important function of the DEGs in the biological process of nervous system activity, we further intersected 14 hub genes and 24 nervous system-specific expressed genes and identified five nervous system-specific expressed hub genes, including APP, AKT1, LRRK2, PSEN1, and SLCO1A2 (Table 3, in bold).

**Table 3:** Hub genes determined using cytoscape plug-in cytoHubba by five algorithms

Gene	Description	Log2FC	Adjust *p* value	Regulation
**AKT1**	Serine/threonine kinase 1	4.183	0.007	Up
**PSEN1**	Presenilin 1	5.0827	0.030	Up
**APP**	Amyloid beta precursor protein	5.3519	0.021	Up
**LRRK2**	Leucine rich repeat kinase 2	2.8715	0.029	Up
**SLCO1A2**	Solute carrier organic anion transporter family member 1A2	3.9143	0.030	Up
MYOG	Myogenin	2.2627	0.041	Up
MDM2	MDM2 proto-oncogene	0.4255	0.018	Up
LIG4	DNA ligase 4	2.7041	0.048	Up
XRCC4	X-ray repair cross complementing 4	2.8248	0.034	Up
CCNB1	Cyclin B1	3.1106	0.031	Up
ATF3	Activating transcription factor 3	2.7574	0.018	Up
JAG1	Jagged canonical Notch ligand 1	2.2920	0.028	Up
RAD9A	RAD9 checkpoint clamp component A	− 4.7490	0.019	Down
AHR	Aryl hydrocarbon receptor	− 1.6973	0.035	Down

*FC* fold change

### Construction of mRNA–miRNA coexpression networks

**Fig. 5:**
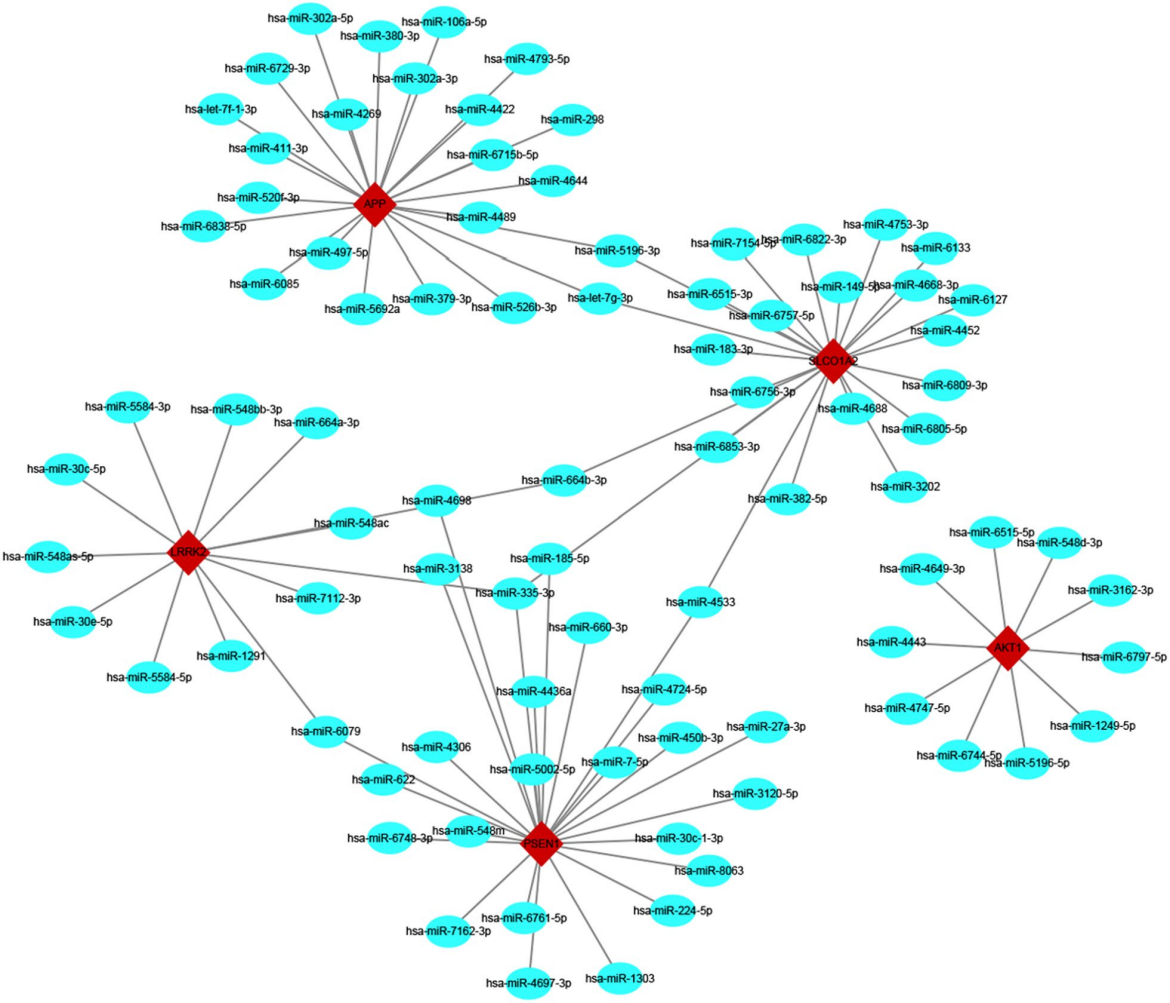
Construction of mRNA–miRNA coexpression networks. A co-expressed network of mRNAs and target miRNAs. The mRNA–miRNA co-expressed network was constructed by Cytoscape including 92 nodes and 94 degrees. Red diamonds represent five hub genes and blue circles represent the target miRNAs

MiRNAs have been reported to regulate gene levels by binding the 5′ or 3′ UTR of the target mRNA and play an important role in neurological disorder development. Therefore, we predicted the target miRNAs of 5 nervous system-specific expressed hub genes using five online miRNA databases and identified 87 target miRNAs and 94 mRNA–miRNA pairs. Finally, we constructed a coexpression network of mRNAs and miRNAs by Cytoscape, which comprised 92 nodes and 94 edges (Fig. 5).

### Verification of five nervous system-specific expressed hub genes in three GEO datasets

**Fig. 6:**
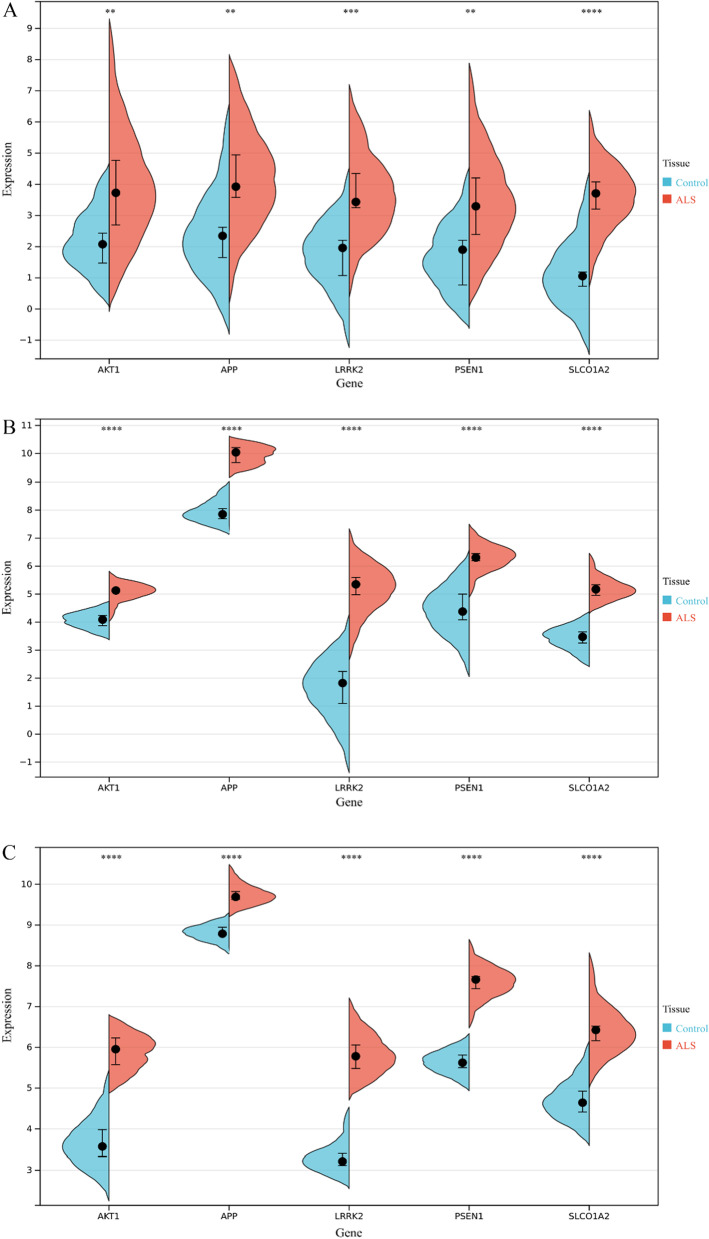
Verification of five nervous system-specific expressed hub genes in three GEO datasets. **A**–**C** Five hub genes are significantly upregulated in sALS patient samples compared with nonneurological control samples verified by three datasets: GSE4595, GSE26927 and GSE39644, respectively (***p < 0.001, **p < 0.01, *p < 0.05)

Three GEO datasets, namely, GSE4595, GSE26927 and GSE39644, including 29 nonneurological control samples and 31 sALS patient samples, were used to verify the expression levels of 5 nervous system-specific expressed hub genes. The R software ggplot2 package was used to generate a split violin plot, and the differences were analyzed by Student’s t-test. As expected, we found that the mRNA expression levels of the 5 nervous system-specific expressed hub genes in the sALS group were significantly higher than those in the nonneurological control groups (Fig. 6A–C, *p* < 0.01), implying that the 5 hub genes play an important role in sALS development. In addition, we performed GO, KEGG and GSEA enrichment analyses based on five nervous system-specific expressed hub genes. As expected, the enriched gene sets of the DEGs were related to the neurotrophin signaling pathway, glial cell activation, neuron death, axon guidance, regulation of the actin cytoskeleton, etc., which are correlated with sALS progression (Additional file 1: Fig. S1A–G).

### ROC curve and prognosis prediction ability of 5 nervous system-specific expressed hub genes in sALS samples

**Fig. 7:**
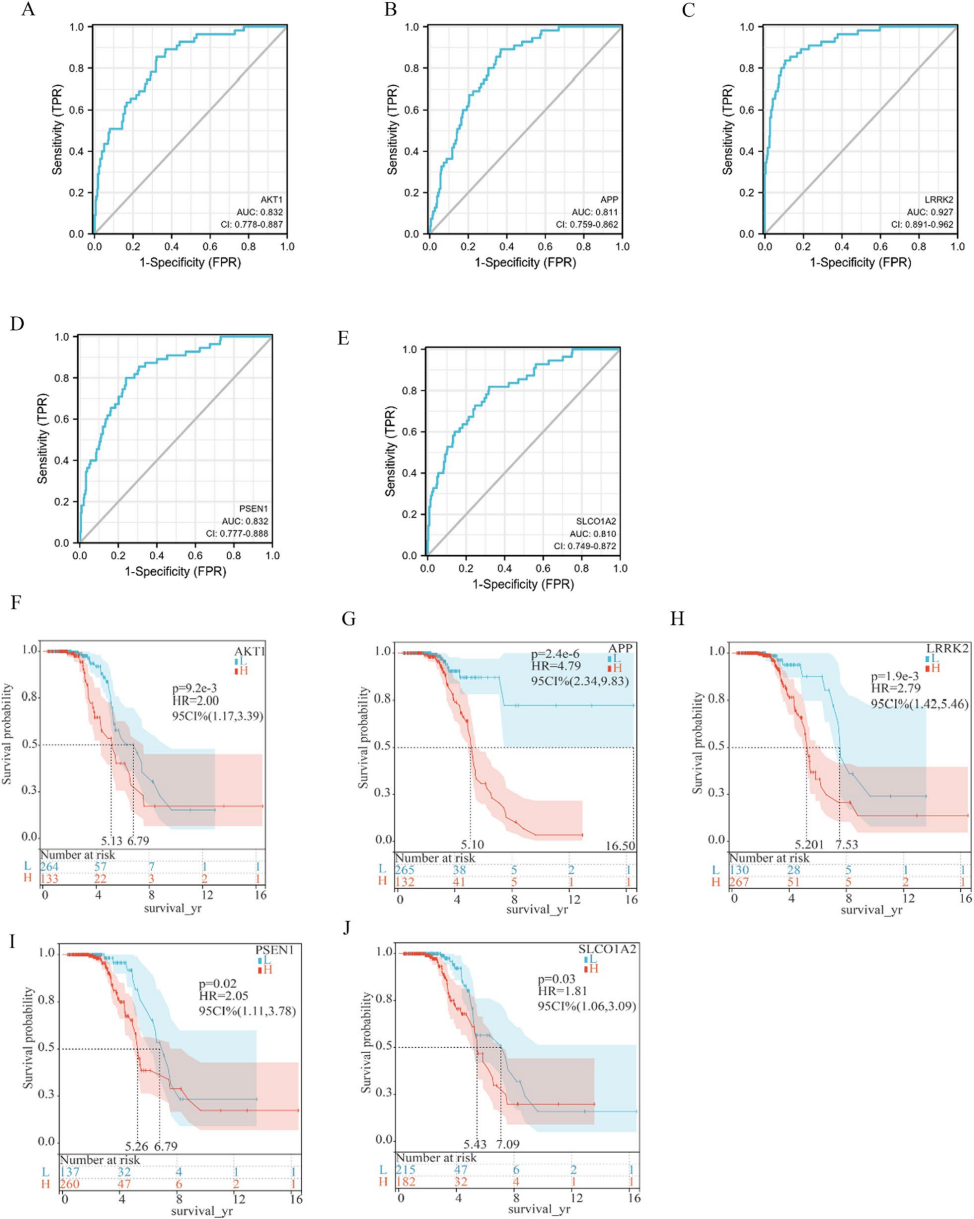
ROC curve and prognosis prediction ability of 5 nervous system-specific expressed hub genes in sALS samples. **A**–**E** ROC curve of 5 nervous system-specific expressed hub genes were constructed by GSE112681 dataset. The AUC of these genes was 0.832 for AKT1, 0.811 for APP, 0.927 for LRRK2, 0.832 for PSEN1, and 0.810 for SLCO1A2. **F**–**J** survival analysis show that high expression of these genes predicted poor prognosis for sALS. **F**–**J** survival analysis based on hub genes expression by Kaplan–Meier method

Since sALS patients are difficult to diagnose in the early stage because of clinical heterogeneity, it is essential to find diagnostic biomarkers to distinguish individuals with sALS from nonneurological people. We used ROC curves to evaluate the diagnostic ability of the 5 hub genes in predicting sALS. Specifically, the expression profiles of 5 hub genes in the GSE112681 dataset of nonneurological control samples and sALS samples were analyzed by the R software pROC package. The ROC curves of these hub genes were drawn, and the area under the curve (AUC) was calculated. The results showed that all hub genes have strong diagnostic value in sALS samples. Our results showed that these 5 hub genes could be biomarkers for sALS diagnosis. LRRK2 had the highest diagnostic ability (AUC: 0.927), while the AUC of the other genes was 0.832 for AKT1, 0.811 for APP, 0.832 for PSEN1, and 0.810 for SLCO1A2 (Fig. 7A–E). Additionally, we evaluated the ability of these genes to predict the prognosis of sALS patients by a survival analysis. A survival curve of the sALS patients based on the expression of the 5 hub genes was drawn by the Kaplan–Meier method. The results showed that sALS patients with high expression levels of AKT1, APP, LRRK2, PSEN1, or SLCO1A2 had a poorer prognosis than those with a low expression of these hub genes (Fig. 7F–J). Our results imply that AKT1, APP, LRRK2, PSEN1 and SLCO1A2 may be biomarkers for the diagnosis and prognosis prediction of sALS patients based on our present samples.

### Construction of mRNA–miRNA–ncRNA coexpression networks

**Fig. 8:**
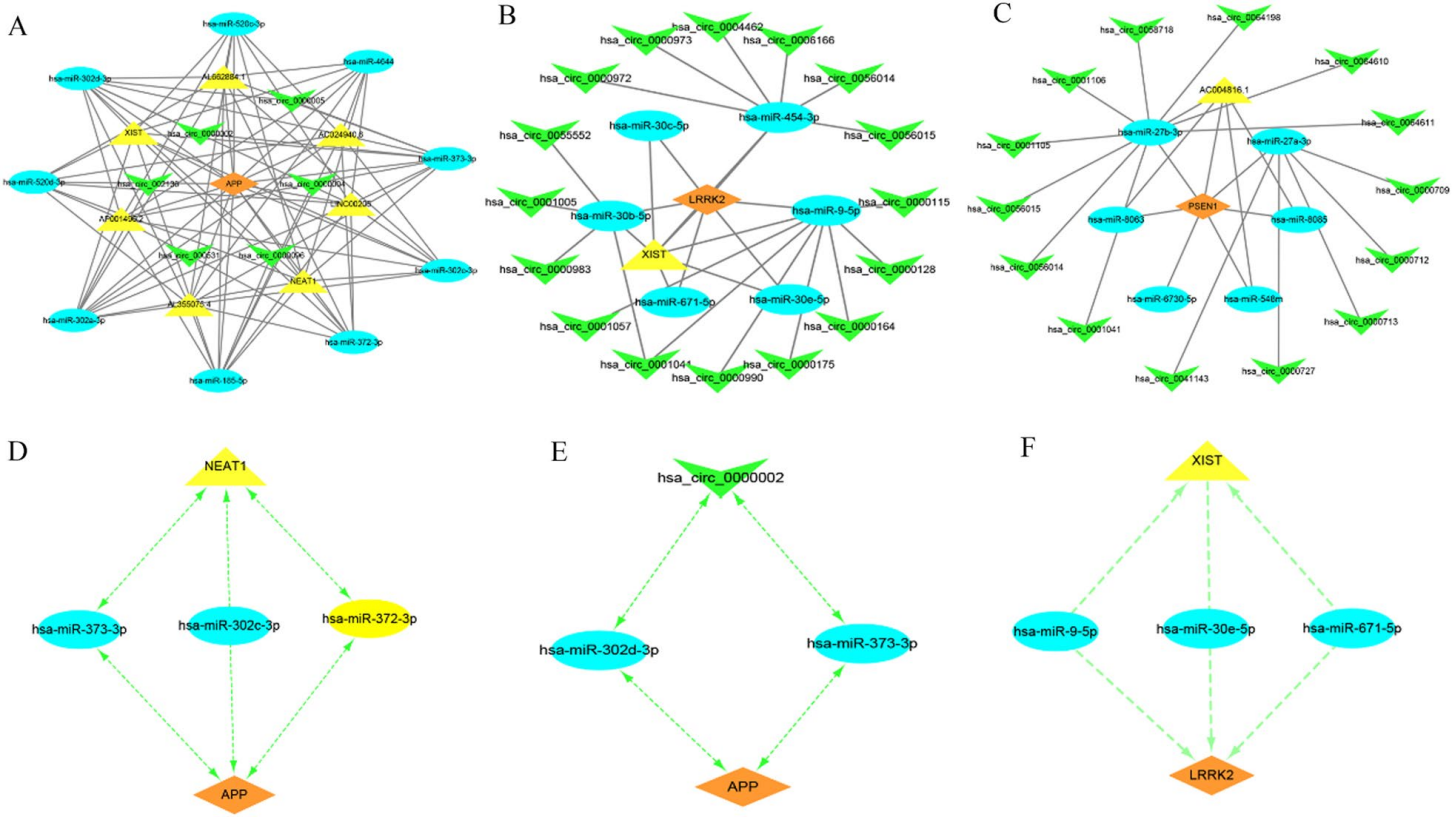
Construction of mRNA–miRNA–ncRNA coexpression networks. **A** CeRNA networks of APP. **B** CeRNA network of LRRK2. **C** CeRNA network of PSEN1. **D** CeRNA network of NEAT1-miR-373-3p/miR-302c-3p/miR-372-3p-APP. **E** ceRNA network of circ_0000002-miR-302d-3p/miR-373-3p-APP. **F** CeRNA network of XIST-miR-9-5p/miR-30e-5p/miR-671-5p. Orange diamonds represent three nervous system-specific expressed hub genes, blue circles represent target miRNAs, yellow triangles represent lncRNA and green V represents the circRNA

MiRNAs can cause posttranscriptional gene silencing by binding mRNAs, while other ncRNAs, such as lncRNAs and circRNAs, can regulate gene expression by competitively binding miRNAs, which is called the ceRNA mechanism. CeRNAs can disable microRNAs by binding microRNA response elements (MRS), which reveal mRNA–miRNA–ncRNA interaction coexpression networks. The existence of microRNA regulatory pathways is of great biological significance. We predicted the circRNAs and lncRNAs that interacted with selected miRNAs by the online database StarBase 3.0. The screening criteria were as follows: (1) mammalian; (2) human h19 genome; (3) and strict stringency (≥ 5) of CLIP-Data with degradome data. NcRNAs present in most selected miRNA predictions were chosen as the predicted circRNAs and lncRNAs. In the predicted results of the StarBase database, a transcript has multiple circRNA shearing sites; thus, the circRNA with the largest number of samples and the highest score in the circBase database was selected as the target circRNA. Finally, we obtained 7 lncRNAs and 5 circRNAs from APP-targeted miRNAs, 1 lncRNA and 16 circRNAs from LRRK2-targeted miRNAs, and 1 lncRNA and 14 circRNAs from PSEN1-targeted miRNAs. Three ceRNA networks were constructed based on the prediction results and visualized by Cytoscape (Fig. 8A–C). Subsequently, we conducted a literature search and selected seven miRNAs and three ncRNAs that were reported to affect neurodegeneration disorder development for further study. We propose that NEAT1-miR-373-3p/miR-302c-3p/miR-372-3p-APP, circ_0000002-miR-302d-3p/miR-373-3p-APP and XIST-miR-9-5p/miR-30e-5p/miR-671-5p might be potential ceRNA regulatory pathways that regulate sALS progression (Fig. 8D–F).

### Identification of the genetic association between 3 SNPs and sALS

**Fig. 9:**
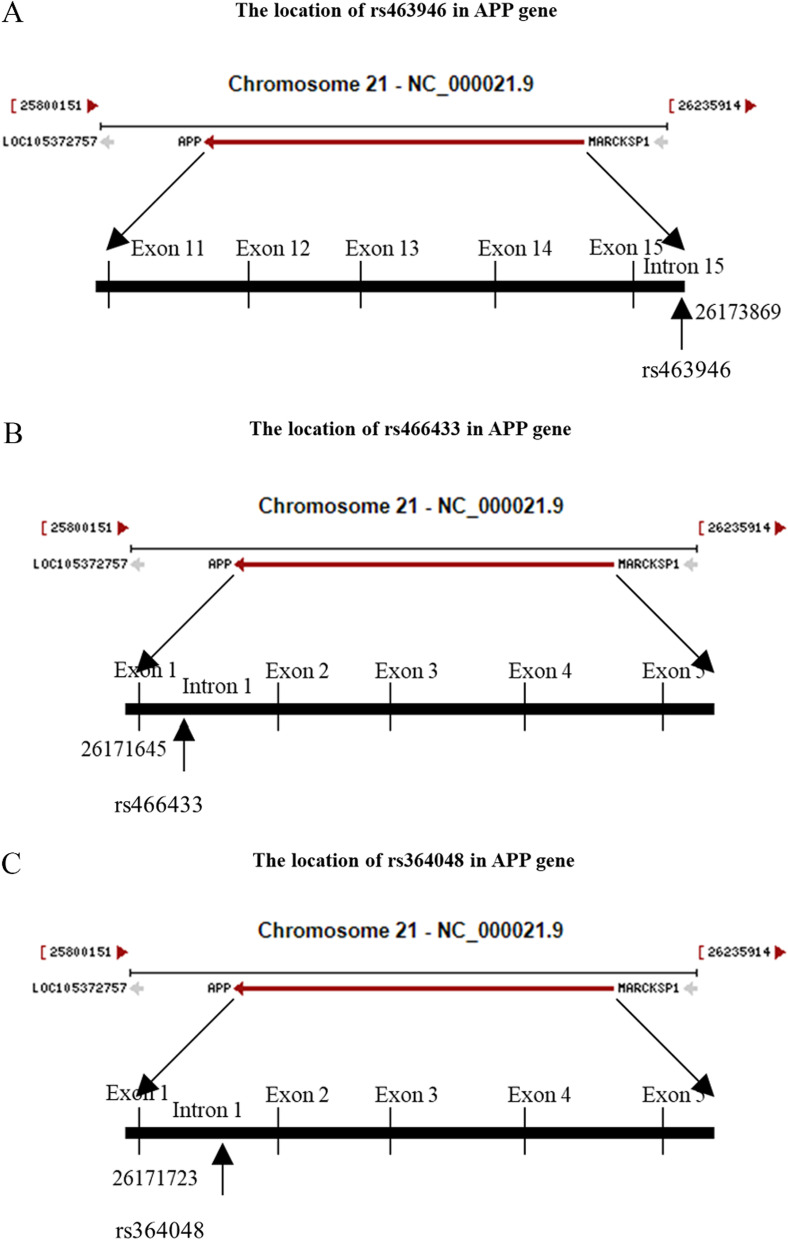
Locations of rs463946, rs466433, rs364048 in the APP gene. **A** The rs463946 is located in intron 15, at position 26,173,869 of the APP gene on chromosome 21. **B** The rs466433 is located in the intron 1, at position 26,171,645 of the APP gene on chromosome 21. **C** The rs364048 is located in intron 1, at position 26,171,723 of the APP gene on chromosome 21

To date, several studies have confirmed that AD (Alzheimer’s disease), PD (Parkinson’s disease), FTD (frontotemporal dementia), PSP (progressive supranuclear palsy) and ALS have similar genetic bases. SNPs of APP (rs463946, rs466433, and rs364048) have been found to be closely related to the incidence of AD in the Chinese Han population. Therefore, we hypothesized that these three SNPs of APP are involved in sALS development, and the locations of the three SNPs of APP are shown in Fig. 9 A-C. All 3 SNPs were intronic polymorphisms, and the minor alleles of rs463946 (OR = 3.000, 95% CI 1.164–7.732, *p* = 0.014), rs466433 (OR = 3.000, 95% CI 1.164–7.732, *p* = 0.014), and rs364048 (OR = 3.000, 95% CI 1.164–7.732, *p* = 0.014) significantly increased the risk of sALS development, suggesting that these three polymorphisms might represent genetic susceptibility factors. Subjects harboring the minor G allele (GG + CG) of rs463946 (*p* = 0.026), minor G allele (GG + AG) of rs466433 (*p* = 0.026) and minor C allele (CC + CT) of rs364048 (*p* = 0.026) showed an increased risk of sALS development compared with those with the other genotypes (Table 4).

**Table 4:** Three SNPs shown the significant at *p* < 0.05 in the study

SNP	Group	Genotypes			Genotypes *p* value	Allele		Allele *p* value	OR (95%)
		GG	CG	CC		G	C		
rs463946	Cases (n = 30)	2	11	17	0.026	15 (25.0%)	45 (75.0%)	0.014	G:3.000 (1.164–7.732)
	Controls (n = 30)	1	3	26		5 (8.3%)	55 (91.7%)		C:0.818 (0.694–0.965)

SNP	Group	Genotypes			Genotypes *p* value	Allele		Allele *p* value	OR (95%)
		GG	AG	AA		G	A		
rs466433	Cases (n = 30)	2	11	17	0.026	15 (25.0%)	45 (75.0%)	0.014	G:3.000 (1.164–7.732)
	Controls (n = 30)	1	3	26		5 (8.3%)	55 (91.7%)		A:0.818 (0.694–0.965)

SNP	Group	Genotypes			Genotypes *p* value	Allele		Allele *p* value	OR (95%)
		GG	CC	CT		C	T		
rs364048	Cases (n = 30)	2	11	17	0.026	15 (25.0%)	45 (75.0%)	0.014	C:3.000 (1.164–7.732)
	Controls (n = 30)	1	3	26		5 (8.3%)	55 (91.7%)		T:0.818 (0.694–0.965)

*SNP* single nucleotide polymorphism, *OR* odds ratio

## Discussion

Amyotrophic lateral sclerosis is a highly fatal neurodegenerative disease, and currently, effective treatment is lacking. The cause of ALS currently remains unknown, although some people have familial disease, which is associated with mutations in genes that perform a wide range of functions [20]. Research investigating the pathogenic factors of sALS is still in a relatively underdeveloped state [21]. Based on the current predicament that sALS is difficult to diagnose due to the lack of biomarkers, new diagnostic biomarkers and prognostic biomarkers of sALS are urgently needed.

In this study, we identified 3 nervous system-specific expressed hub genes, which play an important role in sALS progression. According to the current understanding of the pathogenesis of sALS, axonopathy, aberrant RNA metabolism, nucleocytoplasmic and endosomal transport, oligodendrocyte degeneration, neuroinflammation and mitochondrial dysfunction were reported to be involved in sALS development [22]. The results of our GO, KEGG pathway and GSEA analyses of hub genes functions are consistent with the pathogenesis of sALS, indicating that elucidating the functions of these DEGs in sALS might help us gain a deeper understanding of the pathophysiology of sALS.

The expression levels of three nervous system-specific expressed genes (APP, LRRK2, PSEN1) were validated in three GEO datasets. We further evaluated whether these genes could be used as diagnostic biomarkers of sALS. The ROC curve analysis showed that all these genes have a high diagnostic ability for sALS. Survival analysis confirmed that a high expression of these genes predicted a poor prognosis of sALS, indicating that these genes could also be prognostic biomarkers of sALS.

Thus far, studies have shown that AD, PD, FTD, PSP and ALS have similar genetic bases [23]. With the development of research concerning neurodegenerative diseases, many neurodegenerative diseases are believed to exhibit varying degrees of genetic and pathological overlap, indicating that genes or proteins associated with the pathogenesis of one neurodegeneration disease may also be associated with other diseases. For example, the deletion of the SOD1 gene promotes APP protein oligomerization and memory loss in an AD mouse model, suggesting that SOD1 may be involved in the regulation of APP metabolism in AD patients [24]. The amyloidosis process of APP in the central nervous system may lead to the degeneration of motor neurons. Bryson J B et al. reported that after the knockdown of the APP gene in a SOD1 mouse model, innerve, motor function, viable motor neurons and other disease parameters of the mice were significantly recovered, indicating that APP was involved in the pathogenesis of SOD1-mediated ALS [25]. There are more and more studies on early diagnosis of ALS using plasma/serum or cerebrospinal fluid (CSF). Reports showed that soluble APP fragments and Aβ peptides levels are altered in plasma/serum or CSF form ALS patients, which could serve as the biomarkers of ALS Pathophysiology [26]. Another report also showed that CSF Aβ_42_ increased remarkably in the ALS groups [27]. Our results showed that APP was highly expressed in the sALS samples, which is consistent with reports showing that APP was found in the spinal cord, skin and muscle of ALS patients [28, 29].

Since neurodegenerative diseases, such as AD, PD, FTD, PSP and ALS, share a similar genetic basis and SNPs of APP were reported to be involved in AD progression [30, 31], we investigated the effect of SNPs of APP in sALS patients. The results confirmed that three minor alleles, rs463946, rs466433 and rs364048, increased the risk of sALS development. A possible reason is that the metabolic process of the APP protein is affected by SNPs of APP, which reduces the normal decomposition of the APP protein and leads to the occurrence of sALS. However, more investigations are needed to confirm this hypothesis.

CeRNA networks play an important role in neurodegenerative disease progression. LncRNAs, miRNAs and circRNAs are important component factors in ceRNA networks. In our study, the target miRNAs, target lncRNAs and circRNAs of these miRNAs were predicted for APP, LRRK2 and PSEN1. We identified three important potential RNA regulatory pathways in the pathogenesis of sALS, including NEAT1-miR-373-3p/miR-302c-3p/miR-372-3p-APP, XIST-miR-9-5p/miR-30e-5p/miR-671-5p-LRRK2 and circ_0000002-miR-302d-3p/miR-373-3p-APP.

Our study has some shortcomings. The sample size for testing and validation was relatively small, and the expression of the three hub genes in sALS samples should be further examined using fresh tissue samples. In addition, the diagnostic ability and prognostic ability of the three hub genes of sALS should be further confirmed in prospective cohort studies.

## Conclusions

Our study identified 3 nervous system-specific expressed hub genes, APP, LRRK2 and PSEN1, as potential diagnostic and prognostic biomarkers of sALS, and our results provide new insight into the pathogenesis of sALS at the transcriptome level. We also identified three potential RNA regulatory pathways that affect sALS progression by ceRNA network construction.

## Supplementary Information

**Additional file 1**. Primer sequences genotyped and enrichment analysis of 5 nervous system-specific expressed genes.

## Data Availability

The GEO dataset data used in this study are available in the GEO database (https://www.ncbi.nlm.nih.gov/geo/) with the following data accession identifiers: GSE833, GSE26276, GSE4595, GSE26927, and GSE39644 and GSE112681.

## Abbreviations

ALS: Amyotrophic lateral sclerosis
sALS: Sporadic amyotrophic lateral sclerosis
GEO: Gene expression omnibus
DEGs: Differentially expressed genes
GO: Gene ontology
GSEA: Gene set enrichment analysis
KEGG: Kyoto encyclopedia of genes and genomes
PPI: Protein–protein interaction
MNC: Maximum neighborhood component
MCC: Maximal clique centrality
DMNC: Density of maximum neighborhood component
ceRNA: Competitive endogenous RNA
ROC: Receiver operating characteristic
AUC: Area under the curve
ncRNAs: Noncoding RNAs
