# Supplementary material for: Three nervous system-specific expressed genes are potential biomarkers for the diagnosis of sporadic amyotrophic lateral sclerosis through a bioinformatic analysis
Source: BMC Med Genomics. 2023 Jan 27;16:15. doi: 10.1186/s12920-023-01441-x (PMC9881351; doi:10.1186/s12920-023-01441-x)
Supplement: Supplementary file 1 — Additional file 1. Primer sequences genotyped and enrichment analysis of 5 nervous system-specific expressed genes. [file 12920_2023_1441_MOESM1_ESM.docx]

Additional file 1: Table S1. Primer sequences genotyped by sequenom.

| SNP ID | Forward primer | Reverse primer | Extend primer | Extend product |
| --- | --- | --- | --- | --- |
| rs463946 | CCAGTGACCAAGCAGAATA | GGAAGGAACCAGAAACCA | CTGACTGAGGTTTTCATG  CTACTTCTTCCT | F30CG |
| rs466433 | TGTAACATCCATCTGAGTTG | AGAAAGTGGCATGGCAGAAG | CTGACTGACTGACTGAGTAAATGAGGACTTCTGACCTC | F38AG |
| Rs364048 | GTGTCTGTCCTGAATTATAG | ATATATCTGGGCAGTTCTAGAG | CTGACTGACTGACTGACTGACTGAGCCATGCCACTTTCTCCTGGAT | F46CT |

Additional file 1: Fig. S1


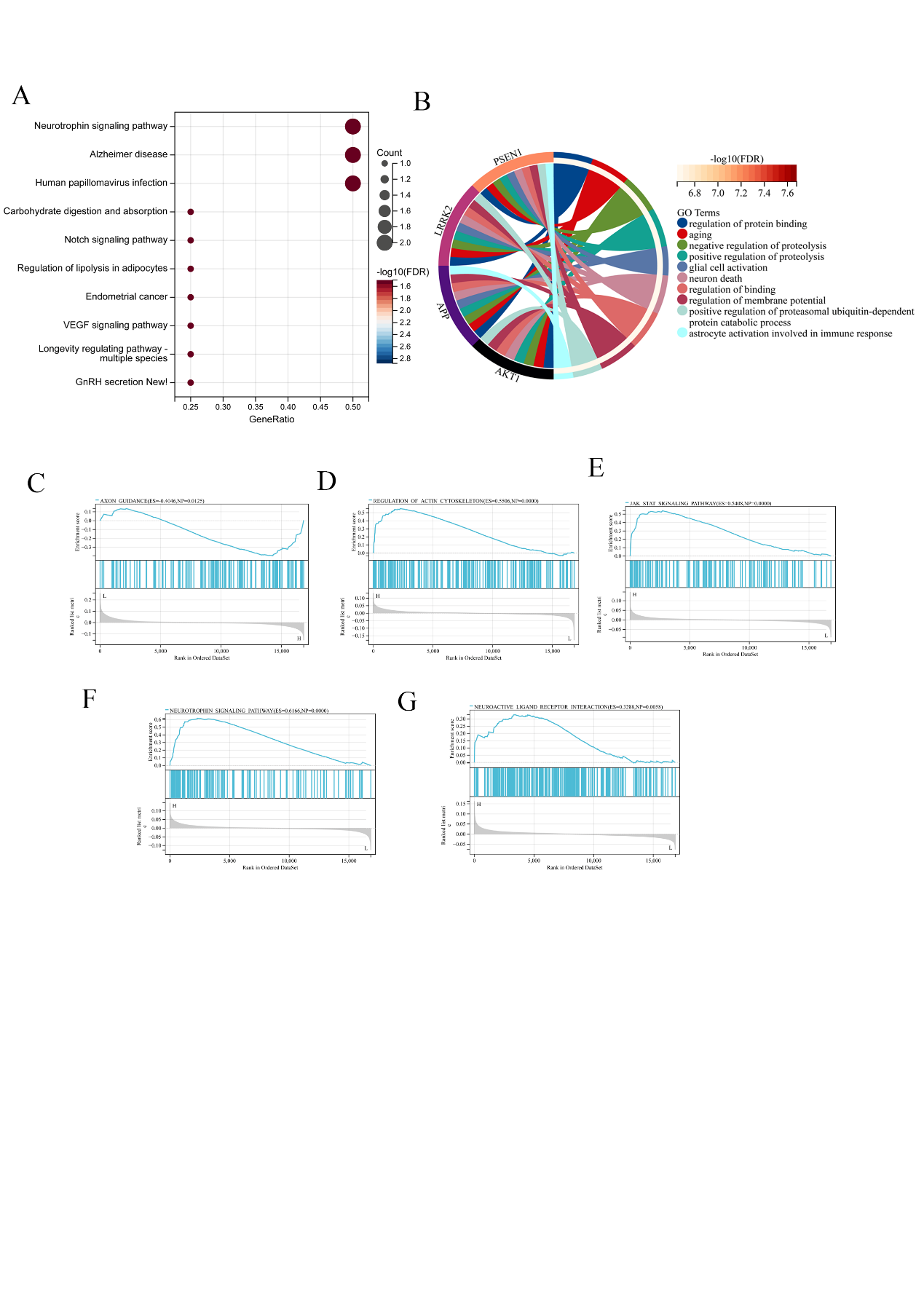


**The enrichment analysis of 5 nervous system-specific expressed genes.**

A: KEGG enrichment of 5 nervous system-specific expressed genes indicated these genes were mainly involved in neurotrophin signaling pathway, Alzheimer's disease and human papillomavirus infection. B: GO analysis showed the relative biological function were involved in regulation of protein binding, aging, negative regulation of proteolysis, positive regulation of proteolysis, glial cell activation, neuron death, regulation of binding, regulation of membrane potential, positive regulation of proteasomal ubiquitin-dependent protein catabolic process and astrocyte activation involved in immune respone. C: GSEA analysis indicated these genes were involved in axon guidance, regulation of actin cytoskeleton, JAK STAT signaling pathway, neurotrophin signaling pathway, neuroactive ligand receptor interaction.
